# Understanding the Impact of Socioeconomic Factors on Early Childhood Development in Marginalised Roma Communities: The Role of Parental Education and Household Equipment

**DOI:** 10.3390/children11060622

**Published:** 2024-05-23

**Authors:** Jana Plavnicka, Shoshana Chovan, Daniela Filakovska Bobakova

**Affiliations:** 1 Department of Health Psychology and Research Methodology, Faculty of Medicine, PJ Safarik University, 041 11 Kosice, Slovakia; shoshana.chovan@student.upjs.sk (S.C.); daniela.filakovska@upjs.sk (D.F.B.); 2 Olomouc University Social Health Institute, Palacky University in Olomouc, 771 11 Olomouc, Czech Republic

**Keywords:** early childhood, disadvantage, marginalised Roma communities, development, parental education, household equipment

## Abstract

This study aimed to explore the effect of socioeconomic disadvantage accumulated in marginalised Roma communities (MRCs) on early childhood development and to assess the role of selected socioeconomic indicators in the association between belonging to MRCs vs. the majority and early childhood development. We obtained cross-sectional data from 232 mother–child dyads from MRCs and the majority population. The differences in early childhood development and background variables between the two groups were tested using chi-square and Mann–Whitney U tests. The moderated mediation was tested using PROCESS Macro in SPSS Model 14 on 5000 bootstrap samples. Statistically significant differences between children from MRCs and the majority were found in terms of maternal age, parental education, household equipment, as well as early childhood development. Household equipment moderated the indirect effect of being from MRCs vs. the majority on early childhood development through parental education. The indirect effect through parental education was high at a low household equipment level, reduced at an average level and non-significant at a high level of household equipment. Our study uncovered disparities in early childhood development between children from MRCs and the majority population. Parental education significantly influenced developmental outcomes, while household equipment mitigated its impact.

## 1. Introduction

The foundation for further cognitive, social-emotional, behavioural, personality, and language development is laid during early childhood, with later impacts on academic and professional trajectories [1]. Early childhood development in this crucial period reflects the maturation of the developing brain in interaction with the environment [2,3]. However, contextual factors such as poverty, segregation, environmental hazards, and a prevalence of adverse childhood experiences pose significant challenges to optimal development [4]. The effects of poverty during early childhood can have long-lasting consequences for development, often more severe than if experienced later in life [5]. Many health problems in adulthood are developmental problems that originate from challenges in childhood and are related to economic disadvantage, discrimination, or neglect [6].

Marginalised Roma communities (MRCs) are among the most disadvantaged and underserved communities in the EU. People living in MRCs face discrimination and socioeconomic deprivation and have limited access to education, labour markets and various services [7]. Out of approximately 440,000 Roma living in Slovakia, more than half live in so-called marginalised Roma communities (MRCs) [8], in which 87% of households are at risk of poverty, 52% face severe material deprivation, and 58% have inadequate housing. While most non-Roma households have essential equipment such as a connection to the electricity network, water supply and sewerage, a bathroom or shower and a flush toilet, a smaller proportion of households from MRCs have this equipment. Approximately one-third of households in MRCs lack showers, flush toilets, and sewerage [9]. The poor living conditions in MRCs can also be observed in other indicators, such as the availability of drinking water. Approximately 22% of the Roma population in Slovakia has no access to drinking water in the household [10].

In addition to addressing socioeconomic factors, it is essential to understand the sociocultural values in MRCs, including the characteristics of cultural practices and family dynamics. Families from MRCs are often based on a strong family unit, family and community upbringing, which stems from their conservative traditions and life values [11]. These cultural practices are an essential part of the Roma community’s identity and sense of belonging to the community [12]. Family structure and family members’ roles are important for maintaining cultural traditions in MRCs. Women are predominantly responsible for childcare and household care [13].

Children from MRCs in Slovakia currently face many risks of disadvantage at the same time. Poverty is linked to a number of factors, both individually and collectively. Its impact on health begins through biological pathways at an early age, representing a vulnerable period. Poverty, along with low levels of education, affects children’s health through both immunological and neuroendocrine stress pathways [14]. Anasuri [15] also highlights the influence of individual and socio-contextual factors, emphasising that families organise their activities in the context of the available socioeconomic resources, cultural values, and community norms. In terms of optimal child development, the concept of the toxic stress of poverty and disadvantage is useful in relation to understanding the mechanisms linking housing to biophysiological responses to stress [16]. Living conditions associated with poverty impede healthy physical and cognitive development [17]. In addition, poverty results in increased levels of parental stress and subsequent low levels of positive stimulation provided to children [18]. Quality home care for child development, characterised by cognitive stimulation, sensitive nurturing, access to educational materials, and positive methods of discipline, plays a pivotal role in a child’s overall development. Such care is essential to reduce the negative impacts of poverty on early childhood development and subsequent life outcomes [19,20]. Children from MRCs are typically exposed to different stimuli than children from the majority population. Differences in the types and quality of these stimuli may result in the acquisition of different skills and influence the achievement of developmental goals essential for successful learning [21]. When contextual characteristics do not allow for optimal development and the fulfilment of developmental potential, the consequences carry over into later life and can lead to educational and labour market underperformance, ultimately resulting in a poorer quality of life [22].

The conceptual framework on the social determinants of health [23] emphasises health as a topic of social justice and captures the complexity and interconnections of structural and intermediary determinants of health and well-being. Since early childhood development is crucial for future health and well-being, this framework and its components are highly relevant to the conceptualisation of research in this area. Factors such as ethnicity, education and household equipment represent key indicators of socioeconomic position and material circumstances, which are both integral components within the framework [23]. Education is considered a strong determinant of future employment and income. At the same time, one of the most influential factors and indicators of poverty is parental education, which determines not only the material circumstances of the family (e.g., quality of housing or food) but also their health literacy and social capital [23]. Poverty and disadvantage accumulated in MRCs, as described above, thus influence parents’ ability to provide their children with appropriate conditions for healthy early childhood development, not only in terms of the physical environment but also the environment of relationships or optimal nutrition, which significantly impacts healthy development [22].

No research has been carried out so far in Slovakia on the topic of early childhood development of children from MRCs. Generally, children from MRCs are under-represented in research focusing on early childhood development, and the evidence in this area is limited, even though MRCs can be found not only in Slovakia but across Europe, with the largest communities residing in the Central and Eastern European regions. In addition to the lack of relevant research, very little of it has systematically addressed the impact of socioeconomic disadvantage on early childhood development and it mostly focuses on older children. Studies focusing on socioeconomic disparities in early childhood development used mothers’ or parental education as the only indicator of socioeconomic status [24,25,26,27]. Studies such as the one by Gao et al. [28], using other indicators of socioeconomic status, are rare. For these reasons, our study focused specifically on ascertaining the impact of multiple socioeconomic indicators of disadvantage accumulated in MRCs on early childhood development. Understanding the specific roles of selected socioeconomic factors could help shape policies targeting healthy early childhood development in disadvantaged communities.

The aim of our study was to explore the effect of socioeconomic disadvantage accumulated in MRCs on early childhood development. Next, we aimed to assess the role of selected socioeconomic indicators in the association between belonging to MRCs vs. the majority and early childhood development.

## 2. Materials and Methods

### 2.1. Sample and Procedure

We used data from the first wave of the longitudinal RomaREACH study conducted in the Kosice and Presov regions, where the highest concentration of people living in MRCs can be found. The data were collected in 2021–2022 from 232 mothers with children aged 12–21 months from MRCs and the majority population. The criteria for participant selection ensured that individuals possessed the adequate cognitive capacity to comprehend the questionnaire and that their children were born without premature complications. Our primary recruitment strategy involved paediatricians who recruited respondents during the 10th mandatory preventive check-ups, scheduled between 15 and 18 months of age. Paediatricians were enlisted within both the catchment areas for MRCs and outside areas to recruit participants from both MRCs and the majority population. However, challenges arose during the COVID-19 pandemic, causing a strain on primary paediatric care and limiting recruitment opportunities. To mitigate the impact of the COVID-19 pandemic on recruitment, we also engaged Roma health mediators and social workers, who are trusted within the community, to reach out to potential participants. Given the challenging context of the pandemic and the under-representation of MRCs in research, this multi-channel recruitment strategy was designed to ensure a representative sample. Leveraging Roma health mediators and social workers, who hold the trust of community members, facilitated the recruitment of respondents in MRCs and ensured a sufficient sample size. This approach was particularly crucial given the difficulty of engaging the population from MRCs, which is often under-represented in research efforts. To pursue the recruitment of mothers from the majority population, respondents were also addressed via parental groups on social media.

The data were collected in the cooperating outpatient departments, community centres, or respondents’ households. The mothers from the majority population filled out the questionnaires independently. To cope with the low literacy among the mothers from MRCs, the assistance of the researchers was offered, and the method of assisted self-administered interviews was applied.

### 2.2. Measures

Socioeconomic measures used in the RomaREACH study have been utilised in previous research involving MRCs to assess poverty levels in the lower socioeconomic strata, a dimension not adequately captured solely through educational attainment [29]. Before data collection, a pilot study was conducted with 405 mothers from MRCs and the majority population. A subset of 30 mothers provided feedback on any challenging survey items encountered, leading to necessary adjustments aimed at improving the items’ clarity while retaining the intended meaning.

Sociographic mapping conducted in 2019, as reported in the Atlas of Roma Communities [8], was used to address belonging to a marginalised Roma community based on the place of residence. Respondents were also asked whether their closest neighbours are mostly Roma to verify their place of residence.

Parental education [29] was assessed by the question: “What education did you/your husband or partner complete? (Elementary school—finished or unfinished, Apprentice school, Secondary school, University)”. The sum score of the maternal and paternal educational levels was computed.

Mothers’ marital status was assessed using the following response options: single/married/widow/divorced/in a partnership.

We asked about the number of children using the following open-ended question: “How many children do you have?”

Overcrowdedness was assessed as the number of persons per room. We asked how many children and adults live in the household and how many rooms (living rooms, bedrooms) there are.

We asked whether the child attends daycare using the question, “Does your child attend daycare?” with the responses of yes/no.

Household equipment was regarded as a sum score of the available amenities (cold running water, hot running water, working flushing toilet, working bathroom or shower, electricity), with higher scores indicating better equipment [29].

We assessed early childhood development using the long form (108 items) of the Caregiver-Reported Early Development Instrument (CREDI), containing the domains of cognitive, language, motor, and socioemotional development. The CREDI is an internationally used questionnaire with good psychometric characteristics developed to measure early development in children up to 3 years of age, which is suitable for culturally diverse and low-resource settings [24,30]. For computing the overall development score, we used the CREDI scoring app (https://credi.shinyapps.io/Scoring_App/; accessed on 20 February 2023), which uses multidimensional domain loadings and allows individual items to contribute information about multiple domains of development. The statistical analyses used the raw scaled score as the outcome [30].

### 2.3. Statistical Analyses

The Mann–Whitney U test was used to describe the differences between the two groups (MRCs vs. majority population) for the not normally distributed continuous variables. A chi-square test was used for the dichotomous variables. Next, we explored the association of belonging to MRCs vs. the majority and other possible predictors of early childhood development. We used linear regression on 1000 bootstrapped samples. This approach was chosen due to the non-normal distribution of the model residuals. Finally, we conducted mediation analyses to assess whether parental education mediates the differences in early childhood development between the two groups of children and whether house equipment moderates the mediation pathway. Statistical analyses were performed using IBM SPSS 23 for Windows. Moderated mediation was tested using PROCESS Macro in SPSS Model 14 on 5000 bootstrap samples.

## 3. Results

**Figure 1:**
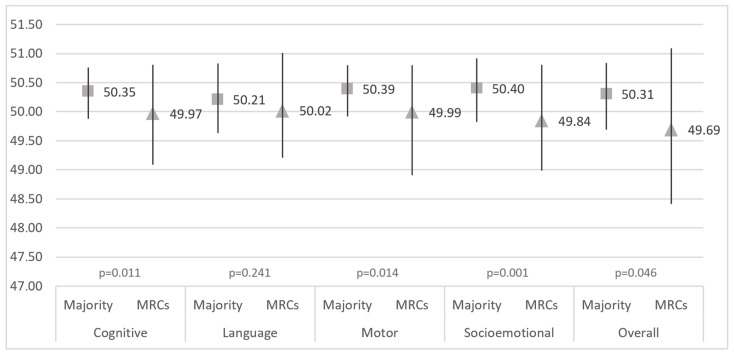
Differences in the cognitive, language, motor and socioemotional domains and overall early childhood development (CREDI questionnaire) between children from MRCs and the majority population (median and interquartile range).

Demographic and socioeconomic descriptions of the sample can be found in Table 1. Statistically significant differences between children from MRCs and the majority can be seen in terms of maternal age, parental education, marital status, number of children, overcrowdedness, household equipment, as well as early childhood development (Table 1). No mothers reported the widowed or divorced marital status. Differences in the cognitive, language, motor and socioemotional domains, as well as in overall early childhood development, between the two groups are shown in Figure 1. All of the observed differences are significant except for the differences in the language domain. Belonging to MRCs vs. the majority and parental education were found to be associated with early childhood development (Table 2).

**Figure 2:**
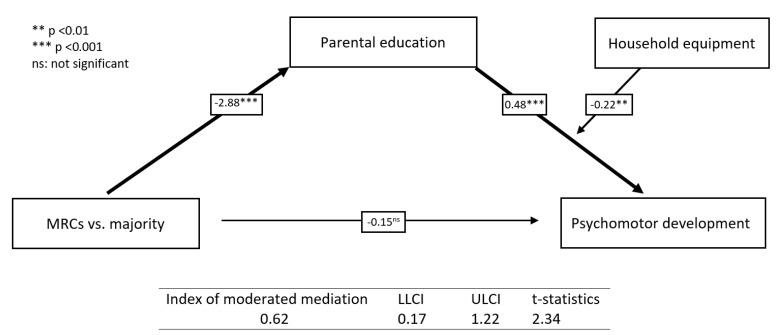
Conditional indirect effect of MRCs vs. the majority on early childhood development mediated through parental education.

In the mediation analysis (Figure 2), we found that the direct effect of being from MRCs vs. the majority on early childhood development was insignificant in the presence of the mediator—parental education. The indirect effect of being from MRCs vs. the majority on early childhood development through parental education was significant, suggesting full mediation. The indirect effect was significant at a low and average level of the moderator—household equipment (Table 3). The post hoc power analyses showed that with a sample size of 232, the effect size calculated for the final moderated mediation model is f^2^ = 0.093, which with α err prob = 0.05 leads to high power (1-β err prob) = 0.98.

**Figure 3:**
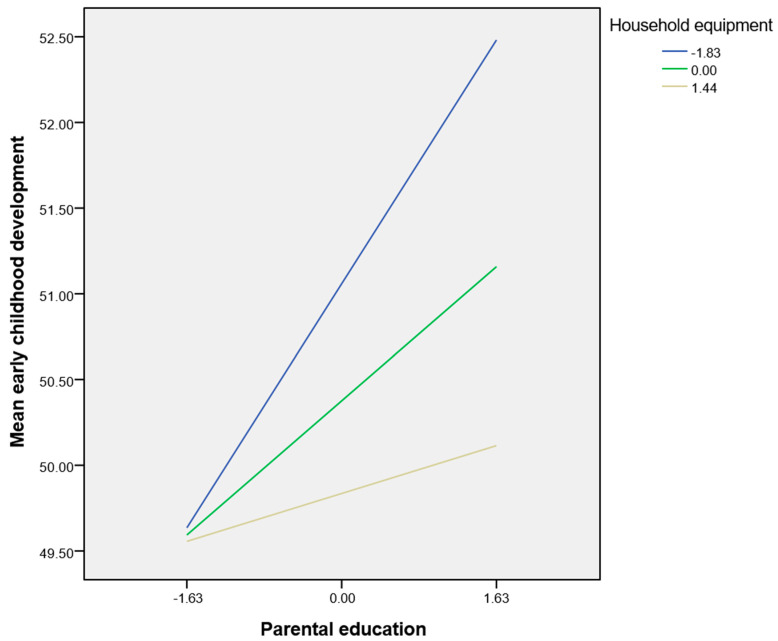
Indirect effect of parental education on early childhood development at low, average and high levels of household equipment.

The index of moderated mediation was 0.62 (CI: 0.17–1.22; t = 2.34). Household equipment moderated the indirect effect of being from MRCs vs. the majority on early childhood development through parental education. This means that household equipment serves as a significant moderator in the pathway linking MRCs vs. the majority to early childhood development, operating through parental education as a mediator. As depicted in Figure 2, the graph illustrates varying gradients across different levels of household equipment, indicating distinct patterns in the indirect effect. The graph (Figure 3) shows a steeper gradient for low and average levels of household equipment. The conditional indirect effect shows that the indirect effect through parental education is high at a low household equipment level, reduced at an average household equipment level and further reduced to a non-significant level at a high level of household equipment (Figure 3, Table 3). Thus, at lower levels of household equipment, the indirect effect through parental education is observed to be substantial, suggesting a significant mediating role. However, as the household equipment levels increase from low to average and subsequently to high, the indirect effect progressively diminishes. This reduction culminates in a non-significant indirect effect at higher levels of household equipment, indicating a diminishing mediating effect of parental education. Household equipment acts as a moderator in the relationship between an MRC background and early childhood development, with the indirect effect through parental education being fully mediated.

## 4. Discussion

The purpose of this research was to explore how socioeconomic disadvantage accumulated in MRCs affects early childhood development and to assess the role of selected socioeconomic indicators in the association between belonging to MRCs vs. the majority and early childhood development. The results showed that parental education mediates the developmental differences between children from MRCs and the majority. Household equipment weakens the relationship between parental education and early childhood development.

The study’s results showed developmental differences between children from MRCs and the majority population. Most of the children included in our study were between 15 and 17 months old, and the developmental delays identified are in line with rare data on early childhood development in disadvantaged Roma children that point to delayed development at 35–59 months of age [24]. Studies that have included children outside MRCs living in socioeconomic deprivation in different countries show similar results [31,32]. It can be assumed that the differences are even more pronounced at later developmental stages [33]. Development at the age of the children, such as in our sample, is largely influenced by biological maturation [34]. However, as children grow older, the range of skills will naturally increase, and there are likely to be more skills in which children from MRCs and the majority will differ. Children acquire skills relevant to development in their setting, and the environment itself is not necessarily less stimulating; it may just differ in the types and quality of the stimuli available. However, we can expect that if we focus on developing skills that predict children’s success in the education system later in life (such as drawing, vocabulary, colour recognition, etc.), children from MRCs will show more significant differences in development due to differential stimulation. Nevertheless, the observed differences in children’s development at this age likely result from the complexity of interrelated factors related to the disadvantaged environment.

We found that the educational level of parents from MRCs is significantly lower than that of the majority population and is associated with worse developmental outcomes in children. Several studies confirmed parental (or maternal) education to be associated with children’s early development or specific domains of development to a certain level [25,26,35,36]. Parental education, occupation and income are highly interrelated components of socioeconomic position [23], which might influence children’s developmental outcomes via different mechanisms [36,37,38]. One of the explanations is that parents’ cognitive abilities and educational attainment might impact the quality of the home environment and parent–child interactions, which are reflected in developmental outcomes. The study by Klein and Kuhhirt [27], focusing on cognitive development using a multigenerational design, suggests that parental cognitive ability accounts for more than half of the association between parental education and children’s outcomes. In terms of generational poverty, which is common in MRCs, it is likely that not only is poverty passed from one generation to another, but also that the interplay of factors contributes to developmental outcomes through complex mechanisms.

Our results indicate that parental education mediates the relationship between MRCs vs. the majority and early childhood development. This suggests that differences in parental education levels fully explain the relationship between MRCs vs. the majority and early childhood development. Parents in MRCs have significantly lower educational levels, which is associated with worse developmental outcomes in children. Similar associations between low educational levels and poorer outcomes in children were found by several studies focusing on children from other ethnic backgrounds [24,25,26,27]. Moreover, exploring the mediating pathways explaining the differences in early childhood development between children from MRCs and the majority population aligns with previous research emphasising the importance of considering mediating mechanisms in analysing the relationship between poverty and children’s developmental outcomes [39]. As parents play a crucial role in children’s early stimulation and development, their level of education and ability to provide support and stimulation may influence various aspects of early childhood development. Mothers with higher educational attainment dedicate more time to nurturing and engaging with their children through essential caregiving, stimulating activities and interactive play than those with lower education levels [15,40]. Due to financial constraints and a lack of information about their importance, parents from MRCs might have limited access to educational materials such as books, toys and interactive resources. They often face financial difficulties and poor living conditions, which significantly limit their ability to provide a stimulating environment for their children [15]. Moreover, access to education supporting parenting skills is severely limited in MRCs compared to the majority, which limits the opportunities for parents from MRCs to gain knowledge and awareness of approaches supporting the healthy development of children.

Although we did not find a direct association between household equipment and early childhood development, our results suggest that household equipment moderates the indirect effect of being from an MRC on early childhood development through parental education. At lower levels of household equipment, the indirect effect is substantial, suggesting a significant mediating role. However, as household equipment levels increase, this indirect effect diminishes. Thus, the presence of basic amenities in the household, such as those we focused on (cold and warm water, toilet, bathroom, and electricity), plays an important role in early childhood development, which is in line with previous studies [28]. Our findings underscore the importance of socioeconomic factors in early childhood development and highlight the need for targeted interventions in low-resource settings. The framework suggested by Dunn [15] describing the specific attributes of housing and their impact on healthy child development, particularly through the lens of the toxic stress of poverty and disadvantage, highlights the critical link between housing conditions and biological responses to stress. Moreover, with unmet basic needs, parents raising their children in poverty face many challenges that affect their ability to provide a nurturing and stimulating environment for their children [41].

The strength of this research lies in the fact that it is the first study to examine early childhood development in children from MRCs, trying to uncover the complex consequences of poverty and disadvantage for early childhood outcomes. However, our study is constrained by its sample size and cross-sectional design, limiting the statistical power and the ability to draw causal conclusions and long-term implications. While we can identify associations between variables, we cannot determine the directionality or causality of these relationships. While efforts were made to mitigate biases, such as providing a conducive environment for data collection, challenges in participant recruitment and data collection methods may affect the representativeness of our findings. The use of multiple recruitment channels, including social media, may have introduced selection bias, as individuals who respond to online recruitment calls may differ systematically from those who do not. Despite the use of community intermediaries, engaging MRCs remained challenging, and some individuals may still be under-represented in the sample despite efforts to recruit them. The research sample does not include Roma mothers and children of higher socioeconomic status who live outside MRCs and mothers from the majority population who live at a disadvantage comparable to that of mothers from MRCs. Thus, the research sample includes two socioeconomically distinct groups on opposite sides of the spectrum—mothers with children from MRCs who are living in poverty and from the majority population with middle to high socioeconomic status. While this dichotomy provides valuable insights, it also limits the generalizability of our findings. Given that the Roma represent a rather heterogeneous ethnic group in terms of the level of inclusion within societies and living conditions [42], the generalizability of the research results is limited to Roma living in disadvantaged communities characterised by spatial and social distance from the majority population. Another potential source of bias is self-report bias. The participants’ responses were based on self-reported data, which can be influenced by social desirability, recall accuracy, and personal perceptions.

These limitations should be considered when interpreting our findings. The potential biases and the constraints of the cross-sectional design suggest that our results should be considered exploratory. They highlight areas where further investigation is needed rather than providing definitive answers. Implications for further research are thus related to including a larger and more representative sample on the continuum of the socioeconomic spectrum to ensure the representation of different socioeconomic categories inside and outside of MRCs. This would allow for a better understanding of the differences in the impact of disadvantage, ethnicity and the home parenting environment on early childhood development. Research indicates that some adverse effects can be lessened or even reversed with proper support, especially during childhood [15]. Further research should also address the effectiveness of interventions concerning housing and early childhood education and care services, taking into account that interventions addressing social determinants of health might not significantly improve an individual’s outcomes due to the cumulating effect of disadvantage in generational poverty, leaving a lasting mark and causing biological influences to be passed down through generations.

Despite these limitations, our results might have implications for policy and practice. The results of the research have shown that disadvantaged children from MRCs are lagging behind in development compared to the majority at the age of 12–21 months. Parental education and household equipment play an essential role in the observed developmental differences. Households struggling with a lack of basic amenities, having limited access to education and employment that would enable them to provide the necessary resources, have limited ability to provide their children with the nurturing and stimulating environment essential for optimal development. Our findings suggest that the availability of basic household amenities plays a crucial role in reducing the influence of parental education on child development. It is, therefore, vital to ensure the basic housing needs of parents in MRCs are met, which could mitigate the negative effect of low educational attainment on the development of children. Policies should ensure accessible and affordable housing for families from MRCs, which may include social housing, subsidising rental housing or providing support for programmes to build or renovate housing for low-income families. In this context, it is also important to involve families from MRCs in various community activities and to support the development of community resources that can improve the quality of their housing. One example of good practice in Slovakia is Project Dom.ov, which addresses housing with microloans and self-help construction of family houses [43]. The “Housing First” approach should be accompanied by the provision of educational and counselling services for parents from MRCs, offering opportunities for parents to learn about parenting approaches that support the healthy development of children to raise awareness and enrich the social capital of parents and to help parents cope with the challenges of parenting in contexts of poverty and resource scarcity. According to Yoshikawa, Abera, and Beardsleeve [44], programmes that support parents in caring for their children represent preventive strategies that have the potential to mitigate the long-term negative impact of poverty on children’s developmental outcomes. An example of good practice in Slovakia is the Omama Project [45]. Women from MRCs, known as Omamas, are trained and regularly visit hundreds of families directly in their home environment. They help mothers from MRCs learn about practical support for their children’s development by playing with them in ordinary situations. The initiative offers benefits by employing women who speak their community’s language and have a more profound knowledge of the context of MRCs and the families they work with.

## 5. Conclusions

Our findings revealed disparities in early childhood development between children from MRCs and those from the majority population. These disparities, consistent with the limited existing data on Roma children’s development, suggest delayed progress in early childhood, with the potential for more pronounced differences during later developmental stages. Our findings point to the need to focus beyond the differences observed between the countries when comparing low-, middle- and high-income countries. Inequities existing within the countries affect the most vulnerable minorities and need to be addressed within national policy strategies focusing on modifiable factors that contribute to them.

Parental education emerged as a crucial mediator, with lower education levels correlating with poorer developmental outcomes in children. This underscores the pivotal role of parents in early stimulation and highlights the need for accessible educational resources and support for families in MRCs to foster optimal development in their children. This approach relates not only to the individual development of children but also to the future prosperity of the whole society. Furthermore, our results suggest that household equipment, while not directly linked to developmental outcomes, decreases the impact of parental education on children’s development, emphasising the importance of basic amenities in nurturing environments. Interventions addressing the social determinants of health, particularly in MRCs, should consider the cumulative effects of generational poverty and biological influences while recognising the potential for positive intervention effects, especially during early childhood. These interventions should be evidence-based and aimed at reducing inequalities and improving living conditions for all children, regardless of their ethnic or social background. By addressing these challenges and supporting community-based initiatives, we can work towards promoting equitable developmental opportunities for children in MRCs and informing evidence-based policies and interventions to support their well-being.

## Figures and Tables

**Table 1:** Description of the sample.

	Majority (*n* = 104)		MRCs (*n* = 128)		Total (*n* = 232)		Chi^2^ Test Value	*p*-Value
	N	(%)	N	(%)	N	(%)		
Child’s sex								
Boy	46	44.2	59	46.1	105	45.3	0.08	0.777
Girl	58	55.8	69	53.9	127	54.7		
Marital status								
Single	4	3.8	9	7.0	13	5.6	50.53	<0.001
Married	86	82.7	47	36.7	133	57.3		
In a partnership	14	13.5	72	56.3	86	37.1		
Daycare	4	3.8	2	1.6	6	2.6	1.19	0.276
	Median (IQR)		Median (IQR)		Median (IQR)		Cohen d	
Maternal age (in years)	32.00 (5.00)		24.00 (9.00)		29.00 (10.00)		1.329	<0.001
Age of the children (in months)	16.00 (2.00)		16.00 (2.00)		16.00 (2.00)		0.057	0.655
Education of parents	6.00 (1.00)		2.00 (1.00)		3.50 (3.00)		2.617	<0.001
Number of children	1.00 (3.00)		4.00 (4.00)		2.00 (3.00)		1.238	<0.001
Overcrowdedness	1.00 (0.33)		4.00 (3.50)		1.73 (3.33)		2.617	<0.001
Household equipment	5.00 (0.00)		1.00 (4.00)		5.00 (4.00)		1.646	<0.001
Early childhood development	50.31 (1.16)		49.70 (2.68)		50.12 (1.91)		0.264	0.046

**Table 2:** Association of early childhood development with the determinant variables (crude effects).

	Early Childhood Development B (CI)	*p*-Value
MRCs vs. majority population	−0.46 (−0.81; −0.11)	0.013
Child’s sex	0.17 (−0.22; 0.55)	0.422
Maternal age (in years)	0.03 (−0.00; 0.06)	0.078
Age of the children (in months)	0.50 (−0.13; 1.11)	0.880
Education of parents	0.20 (0.09; 0.32)	0.001
Marital status	−0.12 (−0.25; 0.01)	0.075
Number of children	−0.03 (−0.13; 0.06)	0.500
Overcrowdedness	−0.01 (−0.11; 0.05)	0.742
Household equipment	0.06 (−0.06; 0.19)	0.356

**Table 3:** Conditional indirect effects of MRCs vs. the majority on early childhood development through parental education at low, average and high levels of household equipment.

Level of Household Equipment	Effect	SE	CI Low/High	t-Statistics	*p*-Value
Low	−2.51	0.78	−4.28/−1.17	3.22	<0.001
Average	−1.38	0.42	−2.27/−0.63	3.33	<0.001
High	−0.49	0.40	−1.29/ 0.29	1.23	0.110

## Data Availability

The data presented in this study are available on request from the corresponding author. The data are not publicly available due to privacy and ethical restrictions.

## References

[1] Heckman J., Pinto R., Savelyev P. (2013). Understanding the mechanisms through which an influential early childhood program boosted adult outcomes. Am. Econ. Rev..

[2] François L.L., des Portes V. (2004). The main stages of psychomotor development from 0 to 3 years of age. La Rev. Du Prat..

[3] Cioni G., Sgandurra G. (2013). Normal psychomotor development. Handb. Clin. Neurol..

[4] Ursache A., Noble K.G. (2016). Neurocognitive development in socioeconomic context: Multiple mechanisms and implications for measuring socioeconomic status. Psychophysiology.

[5] Duncan G.J., Magnuson K., Kalil A., Ziol-Guest K. (2012). The Importance of Early Childhood Poverty. Soc. Indic. Res..

[6] Shonkoff J.P., Garner A.S., Siegel B.S., Dobbins M.I., Earls M.F., McGuinn L., Pascoe J., Wood D.L., High P.C., Donoghue E. (2012). The lifelong effects of early childhood adversity and toxic stress. Pediatrics.

[7] European Union Agency for Fundamental Rights (2022). Roma in 10 European Countries: Main Results.

[8] Ministry of Interior of the Slovak Republic Atlas of Roma Communities 2019. http://www.minv.sk/?atlas-romskych-komunit-2019.

[9] Grauzelova T., Markovic F., Vlacuha R., Lorincz M. (2019). EU Income and Living Conditions in Marginalized Roma Communities: Selected Indicators from the EU SILC MRK Survey 2018.

[10] Ravasz Á., Kovács Ľ., Markovič F. (2020). Atlas of Roma Communities 2019.

[11] Krysachenko V. (2015). Gypsies in Ukraine: Modern problems. Political Sci. Bull..

[12] Mrhálek T., Lidová L., Kajanová A. (2015). Hegemony in the Roma family. Neuroendocrinol. Lett..

[13] Szabóné Kármán J., Endrődy O., Svraka B., Lassú Z.F. (2020). The Gypsy/Roma Children, Families. Sokszínű Pedagógia.

[14] Anasuri S. (2017). Children Living in Poverty: Exploring and Understanding Its Developmental Impact. J. Humanit. Soc. Sci..

[15] Dunn J.R. (2020). Housing and healthy child development: Known and potential impacts of interventions. Annu. Rev. Public Health.

[16] Royce J.B. (2021). The Effects of Poverty on Childhood Development. J. Ment. Health Soc. Behav..

[17] Lipina S.J. (2016). The biological side of social determinants: Neural costs of childhood poverty. Prospects.

[18] Tran T.D., Luchters S., Fisher J. (2017). Early childhood development: Impact of national human development, family poverty, parenting practices and access to early childhood education. Child Care Health Dev..

[19] Luby J., Belden A., Botteron K., Marrus N., Harms M.P., Babb C., Nishino T., Barch D. (2013). The effects of poverty on childhood brain development: The mediating effect of caregiving and stressful life events. JAMA Pediatr..

[20] Mačáková S., Heveriová M., Šimková S., Vavrinčík M. (2015). Učme Sa Učiť Sa.

[21] Shonkoff J.P. (2010). Building a New Biodevelopmental Framework to Guide the Future of Early Childhood Policy. Child Dev..

[22] Solar O., Irwin A. (2010). A Conceptual Framework for Action on the Social Determinants of Health.

[23] Čvorović J. (2022). Paternal investment, stepfather presence and early child development and growth among Serbian Roma. Evol. Hum. Sci..

[24] Farah M.J. (2017). The neuroscience of socioeconomic status: Correlates, causes, and consequences. Neuron.

[25] Noble K.G., Engelhardt L.E., Brito N.H., Mack L.J., Nail E.J., Angal J., Barr R., Fifer W.P., Elliott A.J. (2015). Socioeconomic disparities in neurocognitive development in the first two years of life. Dev. Psychobiol..

[26] Klein M., Kühhirt M. (2023). Parental Education and Children’s Cognitive Development: A Prospective Approach.

[27] Gao Y., Zhang L., Kc A., Wang Y., Zou S., Chen C., Huang Y., Mi X., Zhou H. (2021). Housing environment and early childhood development in sub-Saharan Africa: A cross-sectional analysis. PLoS Med..

[28] Madarasová Gecková A., Babinská I., Bobáková D., Dankulincová Veselská Z., Bosáková L., Kolarčik P., Jarčuška P., Pella D., Halánová M. (2014). Socioeconomic Characteristics of the Population Living in Roma Settlements and Their Association with Health and Health-Related Behaviour. Cent. Eur. J. Public Health.

[29] Seiden J., Waldman M., McCoy D.C., Fink G. (2021). Data Management and Scoring Manual 2021.

[30] Lu C., Cuartas J., Fink G., McCoy D., Liu K., Li Z., Dealmans B., Richter L. (2020). Inequalities in early childhood care and development in low/middle-income countries: 2010–2018. BMJ Glob. Health.

[31] Moore T., McDonald M., McHugh-Dillon H. (2015). Evidence Review: Early Childhood Development and the Social Determinants of Health Inequities.

[32] Meriem C., Khaoula M., Ghizlane C., Asmaa M.A., Ahmed A.O. (2020). Early childhood development (0-6 years old) from healthy to pathologic: A review of the literature. Open J. Med. Psychol..

[33] Piccolo L.R., Noble K.G. (2019). Poverty, early experience, and brain development. Handbook of infant Mental Health.

[34] Cuartas J. (2022). The effect of maternal education on parenting and early childhood development: An instrumental variables approach. J. Fam. Psychol..

[35] Duncan G.J., Magnuson K. (2012). Socioeconomic status and cognitive functioning: Moving from correlation to causation. Wiley Interdiscip. Rev. Cogn. Sci..

[36] Davis-Kean P.E., Tighe L.A., Waters N.E. (2021). The role of parent educational attainment in parenting and children’s development. Curr. Dir. Psychol. Sci..

[37] Cano T. (2022). Social class, parenting, and child development: A multidimensional approach. Res. Soc. Stratif. Mobil..

[38] Guo G., Harris K.M. (2000). The mechanisms mediating the effects of poverty on children’s intellectual development. Demography.

[39] McLanahan S. (2004). Diverging destinies: How children are faring under the second demographic transition. Demography.

[40] Van Laer S., Fiľakovská Bobáková D., Kolarcik P., Engel O., Madarasová Gecková A., Reijneveld S.A., de Kroon M.L.A. (2024). Parenting by mothers from marginalised communities and the role of socioeconomic disadvantage: Insights from marginalised Roma communities in Slovakia. Front. Psychol..

[41] Kalil A., Ryan R. (2020). Parenting practices and socioeconomic gaps in childhood outcomes. Futur. Child..

[42] Zachar Podolinská T., Škobla D. (2018). Why labelling matters: On social construction of Roma/Gypsies in Europe. Slovak. Ethnol..

[43] Ondrášiková F., Šiňanská K. (2020). Analysis of Carrying out Social Work in Housing Focused on the Clients from Marginalised Roma Communities.

[44] Yoshikawa H., Aber J.L., Beardslee W.R. (2012). The Effects of Poverty on the Mental, Emotional, and Behavioral Health of Children and Youth: Implications for Prevention. Am. Psychol..

[45] Cesta Von Program Omama 2023. https://cestavon.sk/program-omama/.

